# Multicenter evaluation of the GenomEra SARS-CoV-2 assay kit

**DOI:** 10.1371/journal.pone.0277925

**Published:** 2022-11-28

**Authors:** Mika Lång, Annika Allard, Soile Blomqvist, Irmeli Iranto, Tytti Vuorinen, Antti-Heikki Tapio, Jiri Vainio

**Affiliations:** 1 Fimlab Laboratories, Department of Clinical Microbiology, Tampere, Finland; 2 Department of Clinical Microbiology, Umeå University, Umeå, Sweden; 3 Department of Health Security, Expert Microbiology Unit, Finnish Institute for Health and Welfare, Helsinki, Finland; 4 Department of Clinical Microbiology, Turku University Hospital and Institute of Biomedicine, University of Turku, Turku, Finland; 5 Abacus Diagnostica, Turku, Finland; Cairo University, EGYPT

## Abstract

Severe acute respiratory syndrome coronavirus 2 (SARS-CoV-2) first emerged in late 2019, and quickly spread to every continent causing the global coronavirus disease 2019 (COVID-19) pandemic. Fast propagation of the disease presented numerous challenges to the health care industry in general and especially placed enormous pressure on laboratory testing. Throughout the pandemic, reverse transcription-PCR (RT-PCR)-based nucleic acid amplification tests have been the primary technique to identify acute infections caused by SARS-CoV-2. Since the start of the pandemic, there has been a constantly growing need for accurate and fast tests to enable timely protective and isolation means, as well as rapid therapeutic interventions. Here we present an evaluation of the GenomEra test for SARS-CoV-2. Analytical and clinical performance was evaluated in a multicenter setting with specimens analyzed using standard-of-care (SOC) techniques. Analytical sensitivity was assessed from spiked respiratory swab samples collected into different viral transport media, and in the best performer eSwab, the limit of detection was found to be 239 IU/mL in a heat processed sample. The GenomEra SARS-CoV-2 Assay Kit did not show specificity/cross-reactivity issues with common micro-organisms or other substances commonly found in respiratory specimens when analyzed both *in vitro* and *in silico*. Finally, the clinical performance was assessed in comparison to SOC techniques used at four institutions. Based on the analysis of 274 clinical specimens, the positive agreement of the GenomEra SARS-CoV-2 Assay Kit was 90.7%, and the negative agreement was 100%. The GenomEra SARS-CoV-2 Assay Kit provided accurate detection of SARS-CoV-2 with a short turnaround time in under 90 min.

## Introduction

By February 2022, the severe acute respiratory syndrome coronavirus 2 (SARS-CoV-2), the virus that causes coronavirus disease 2019 (COVID-19), has been verifiably linked to more than 378 million cases and nearly 5.7 million deaths globally [1]. SARS-CoV-2 belongs to the family Coronaviridae within genus Betacoronaviruses, and is one of the seven known human coronaviruses (hCoVs) [2,3]. In recent years, there has been three highly pathogenic and lethal hCoV outbreaks, caused by SARS-CoV, Middle East respiratory syndrome (MERS)-CoV, and the latest SARS-CoV-2 [4]. All three hCoVs infect the lower respiratory tract, causing acute lung injury or respiratory distress syndrome, septic shock, and multi-organ failure, resulting in a high case fatality ratio [4,5]. SARS-CoV was first reported in Foshan, China in 2002, and the MERS-CoV occurred ten years later in Jordan [6,7]. The current outbreak was first reported in Wuhan, China in late 2019, but the detailed route to global pandemic is still partly unknown [8].

The scale of the global pandemic and the high demand for screening of symptomatic individuals caused dramatic and rapid increase in the sample-testing load, which quickly exceeded the laboratory diagnostic capacity. SARS-CoV-2 is a positive-sense single-stranded RNA (+ssRNA) virus, and thus the nucleic acid amplification tests (NAATs) for respiratory tract specimens are the gold standard to diagnose COVID-19 [9,10]. Due to the biosafety risk, viral cultures are not recommended for COVID-19 testing, but serological e.g. antibody-based techniques can be used as supplemental tools [9]. As the antibody response occurs later than a week after infection, the reverse transcription-PCR (RT-PCR)-based NAATs enable acute diagnosis from nasopharyngeal sampling several days earlier in comparison to serological tests [11,12]. On the other hand, at a later phase after infection the detectable viral load decreases in the upper respiratory tract (URT) but remains detectable for a longer period in the lower respiratory tract (LRT) specimens, such as sputum or tracheal aspirates [11,12]. As the aim is to detect viral infections as early as possible, URT specimens, such as nasopharyngeal swabs (NPS) and oropharyngeal swabs (OPS), are the appropriate sampling choice [11,13].

Fast and accurate diagnostic methods can also reduce the risk of SARS-CoV-2 transmission and enable appropriate protective and medical actions. Since the beginning of the pandemic, the number of authorized NAATs, but also other type of tests, has been ever increasing [14]. There has been a common goal to promote clinical testing, and thus the WHO has provided primers for the genes encoding the structural proteins of the viral envelope (Envp) and the nucleocapsid (N), as well as for the RNA-dependent RNA polymerase (RdRp) [9]. RT-PCR-based mass testing is most efficient when performed centralized, but is inevitably prone to delays if disturbances occur in the chain from sampling to result or if unexpectedly high volumes need to be analyzed, thus clogging the testing pipeline. At best, mass testing takes several hours and at worst, several days from sampling to result. To ease these issues and secure testing for the critically ill, laboratories should have alternative methods in place. Decentralized on-demand rapid testing can significantly reduce the time for test results down to 1–2 hours. These rapid RT-PCR tests can be especially beneficial in hospital settings or even within a central laboratory, even if these tests are not scalable to the extent of current mass testing. Rapid testing can occupy a strategic niche in laboratory diagnostics in a complementary manner providing more testing flexibility and faster turnaround times for high priority cases. However, the time gain should not significantly compromise the detection specificity or sensitivity, which in the case of RT-qPCR is often in a range of 1000 cp/mL [15,16].

The GenomEra SARS-CoV-2 Assay Kit (Abacus Diagnostica, Turku, Finland) received CE-IVD status in July 2020. GenomEra SARS-CoV-2 is a multiplex RT-qPCR test for the dual-detection of RdRp and Envp genes, with a sample pretreatment including a single heating step. The GenomEra technology is based on a simple-to-use reagent concept on a low-cost plastic test chip utilizing dry chemistry, and providing results for up to four samples in approximately 70 min. Here, we describe the multicenter evaluation of the analytical and clinical performance of the GenomEra SARS-CoV-2 Assay Kit, executed at four institutions in Northern Europe during May-July 2020.

## Materials and methods

### GenomEra SARS-CoV-2 assay kit

GenomEra® CDX analyzer consists of an integrated thermal cycler and a time-resolved fluorometer, and it is operated via the GenomEra® CDX Software [17]. The GenomEra test consists of three main components: (i) the GenomEra® SARS-CoV-2 chip holder and test chips, containing the dry chemistry real-time RT-PCR reagents, (ii) 1 mL buffer ampoule, and (iii) sample processing control (SPC) tubes, used for sample pretreatment. The GenomEra SARS-CoV-2 assay utilizes real-time RT-PCR and hydrolysis probes to detect SARS-CoV-2 Envp and RdRp genes (Table 1).

**Table 1:** Oligonucleotides used in GenomEra SARS-CoV-2 assay kit.

Oligonucleotide target and function	Oligonucleotide sequence and modifications
**Envp-FWD**	CATCCGGAGTTGTTAATCCAGT
**Envp-REV**	ACAAAGGCACGCTAGTAGTC
**Envp-P**	Red615^a^-CGTCGGTTCATCATAAATTG-MGB^b^-EDQ^c^
**RdRp-FWD**	GTCACGGCCAATGTTAATGC
**RdRp-REV**	TAAATTGCGGACATACTTATCGG
**RdRp-P**	FAM^d^-CTACTGATGGTAACAAA-MGB-EDQ

^a^ Red615: Sulforhodamine 101 acid chloride.

^b^ MGB: Minor Groove Binder.

^c^ EDQ: Eclipse® Dark Quencher.

^d^ FAM: 6-carboxyfluorescein.

The GenomEra SARS-CoV-2 test was used according to the manufacturer’s instructions provided in the package insert. Briefly, the respiratory swab samples collected in a compatible transport media (Copan eSwab™, universal transport medium (UTM®) or 0.9% NaCl i.e. saline), were mixed for 5 s before 50 μL of samples were transferred to SPC tubes, containing dried MS2 bacteriophage. MS2 functions as an internal control to verify the efficacy of the sample preparation and absence of inhibitors in the PCR reaction [18]. Samples were heated for 5 min at 90°C, and thereafter diluted by emptying a 1 mL buffer ampoule into the SPC tube. After mixing for 5 s, 35 μL of pretreated samples were transferred into the test chips, which were automatically and irreversibly sealed by a GenomEra CDX analyzer prior to 70 min assay run. Thereafter, results were automatically displayed in written and numerical form. The test result was considered as positive, if one or both Envp and RdRp were detected.

### Standard-of-care (SOC) SARS-CoV-2 methods

Various reference methods were used at different evaluations Sites (S1 Table). Cepheid Xpert® Xpress SARS-CoV-2 was used at Sites 1,3, and 4, WHO protocol based Envp and/or RdRp gene in-house RT-PCRs were used at Sites 2–4, and finally Abbott RealTime SARS-CoV-2 and Seegene Allplex™ 2019-nCoV Assay assays were used at Site 1 [9,19].

### Analytical performance

The analytical performance of the GenomEra® SARS-CoV-2 Test Kit was evaluated in the means of analytical sensitivity, specificity, reactivity, and potentially interfering substances. The reproducibility, sample matrix effects, and sample stability were also evaluated. Analytical sensitivity of the assay was evaluated with WHO International Standard for SARS-CoV-2 (NIBSC, UK) containing inactivated England/02/2020 isolate spiked into pooled negative nasopharyngeal swabs collected in four transport media, Copan eSwab, UTM, saline, and PBS. Assay was performed with various viral concentrations ranging from 501–501187 IU/mL. Four replicates per each dilution were initially analyzed to determine the limit of detection (LOD) estimates, which were subsequently confirmed by testing an additional 20 replicates. Sample stability was also assessed in these four transport media in duplicates, by incubating untreated low positive patient samples for 96 h or pretreated samples for 8 h at +4°C or RT, respectively. These samples had reference C_T_ values of ~30 when tested with the Xpert Xpress SARS-CoV-2 assay at Site 4.

The analytical specificity of the assay was evaluated by testing various microorganisms commonly encountered in respiratory specimens (S2 Table). Tests related to analytical specificity and reactivity were performed at evaluation Site 2 except for the cross-reactivity of bacterial strains which were tested at Abacus Diagnostica. Bacterial concentration was estimated based on absorbance at 600 nm (> 0.2 AU) and viral concentrations were quantified by PCR. Also, pure isolated nucleic acids were used (S2 Table). Analytical reactivity was analyzed using extracted SARS-CoV-2 RNA from two strains, Finland/1/20/Wuhan/China and Finland/2/20/Milano/Italia, obtained from patient samples. Extracted RNA samples had reference C_T_ values of 30.99 and 30.69, respectively, obtained at Site 2 with in-house E-gene assay. To analyze extracted RNA, 50 μl of PBS was added to the SPC tube, heated for 5 min at 90°C, vortexed for 5 s, after which 5 ul of extracted RNA was added, mixed and pipetted onto the test chip. Additionally, *in silico* strain coverage analyses of the assay kit primers for Envp and RdRp genes of available SARS-CoV-2 (taxid: 2697049) sequences in the GISAID Initiative Database [20] as of January 9th 2022 was performed and the results evaluated. Mutations which occurred in less than 1000 different available sequences were deemed insignificant. *In silico* cross-reactivity analysis was also performed. *In silico* analyses were performed using WHO instructions for IVD tests [21].

A panel of potentially interfering substances (S3 Table), which may be present in respiratory sample matrices, were tested to evaluate their effect on the GenomEra SARS-CoV-2 assay. The list contains endogeneous and exogeneous substances, and common laboratory disinfectants obtained from Tamro (Vantaa, Finland). All substances were added into negative and spiked low positive (2 x LOD) samples in eSwab and tested using the standard GenomEra SARS-CoV-2 assay protocol. Analytical reproducibility was tested at Site 1 and 2. At all Sites, identical samples consisting of one low positive (1.5 x LOD) sample, dilution of inactivated NATtrol SARS-CoV-2 (Zeptometrix, USA) into Copan eSwab, and one negative sample consisting of pooled nasopharyngeal matrix collected into eSwab from healthy donors, were tested over 5 days.

### Clinical performance

Performance characteristics of the GenomEra SARS-CoV-2 assay were evaluated at four institutions (Sites 1–4), using fresh or frozen respiratory swab samples collected in eSwab, UTM or saline (0.9% NaCl). For 25 samples, the exact media was unknown. At all Sites, the performance was compared to a primary SOC comparator method and a confirmatory SOC method was used if discrepant results occurred. In this study, four different SOC methods were employed varying from site to site (S1 Table). A total of 274 samples were analyzed, from which 184 samples were fresh and 90 samples frozen (S4 Table). At Sites 1–4, different numbers of samples were analyzed, 158, 52, 47, and 17, respectively. Finally, inter-assay linear correlation analysis with clinical samples comparing C_T_ values of Envp and RdRp between the GenomEra SARS-CoV-2 assay and the SOC method, Allplex 2019-nCoV assay, was performed using Origin 2016 (OriginLab, Northampton, USA). Intra-assay linear correlation between Envp and RdRp was also analyzed with the GenomEra SARS-CoV-2 assay. As standard, the GenomEra SARS-CoV-2 assay does not report C_T_ values to the user, but, however, we were kindly provided this data by the manufacturer for these analyses.

### Ethical concerns

The study was designed and executed taking into account local legislation and regulations. This study was performed using left-over uncoded specimens, originally collected for other analytical methods, thus causing no additional invasive procedures or ethical concerns.

### Study design

The study was conducted as a retrospective multicenter evaluation using clinical patient samples tested positive or negative for SARS-CoV-2. The GenomEra SARS-CoV-2 Assay Kit was compared to a routine SOC method at each site to determine clinical performance characteristics. A confirmatory SOC method was employed to determine discrepant results. Additional testing with synthetic SARS-CoV-2 RNA, interfering compounds and other micro-organisms was performed to determine analytical performance.

### Data analysis

Data analysis was performed by following the FDA statistical guidance on reporting results from studies evaluating diagnostic tests. Positive (PPA) and negative (NPA) percent agreement levels and 95% confidence intervals (CI) for the GenomEra SARS-CoV-2 test were calculated using MedCalc Software Ltd. (Version 20.010; accessed August 22, 2021). LOD was evaluated according to approved guidelines [22]. Graphical data was evaluated using standard fitting functions on Origin 2016 (OriginLab, Northampton, USA).

## Results

### Analytical performance

Analytical performance of the GenomEra SARS-CoV-2 Test Kit was evaluated in the means of analytical sensitivity, specificity, reactivity, and several other assay parameters to study assay reproducibility and functionality. The analytical sensitivity for viral genomic RNA was monitored using pooled negative nasopharyngeal swabs collected in four different transport media, Copan eSwab, UTM, saline, and PBS. Of the tested media, the best performance in terms of LOD was achieved with eSwab, with 239 IU/mL in the processed sample which corresponds to 8 IU in the final reaction (Table 2). In declining performance order, these figures were 597 IU and 21 IU in UTM, 1790 IU and 63 IU in saline, and 2088 IU and 73 IU in PBS, respectively.

**Table 2:** Limits of detection of the GenomEra SARS-CoV-2 assay using SARS-CoV-2 genomic RNA spiked in nasopharyngeal swabs collected in four transport media.

IU/ml original sample	IU/ml processed sample	IU/reaction	Result (replicates positive)
**eSwab**			
501187	23866	835	4/4
50119	2387	84	4/4
**5012**	**239**	**8**	**23/24**
2506	119	4	10/12
1253	60	2	1/4
501	24	1	1/4
**UTM**			
50119	2387	84	4/4
25059	1193	42	4/4
**12530**	**597**	**21**	**23/24**
6265	298	10	3/4
5012	239	8	2/4
501	24	1	0/4
**saline (0.9% NaCl)**			
50119	2387	84	4/4
**37589**	**1790**	**63**	**23/24**
25059	1193	42	3/4
5012	239	8	3/4
501	24	1	0/4
**PBS**			
501187	23866	835	4/4
50119	2387	84	4/4
**43854**	**2088**	**73**	**23/24**
37589	1790	63	3/4
25059	1193	42	2/4
5012	239	8	1/4
501	24	1	0/4

At the time of analysis in January 2022, about 6.9 million SARS-CoV-2 genome sequences were available in the GISAID database [20]. *In silico* strain coverage analyses of the Envp and RdRp genes revealed two prevalent mutations, first of which resides in the center of the RdRp-R primer target sequence at the nucleotide position 15598 (Wuhan-Hu-1, Genbank accession number: MN908947) and is present in 0.27% of sequence entries. The second prevalent mutation is located on the 3^rd^ nucleotide from 5’-end of the Envp-F target sequence at the nucleotide position 26149 and is present in 1.0% of sequence entries. None of the variants of concern harbor the RdRp mutation and mainly the Gamma variant harbors the Envp mutation. No strains were found to possess mutations in both Envp and RdRp primer target sequences. *In vitro*, extracted SARS-CoV-2 RNAs of geographically different strains Finland/1/20/Wuhan/China and Finland/2/20/Milano/Italia were detected as positive. All micro-organisms other than SARS-CoV-2 were reported as negative in our cross-reactivity analysis, including MERS-CoV and SARS-CoV. Tested viruses, bacteria, and viral RNAs are all commonly found in respiratory samples or related to SARS-CoV-2 (S2 Table). *In silico* cross-reactivity analysis confirmed that there are only a few oligonucleotide hits to non-target sequences with ≥ 80% sequence homology to Envp and RdRp genes, all of which are non-amplifiable [20]. Non-micro-organism and respiratory sample matrix related potentially interfering substances were also tested without any effect on GenomEra SARS-CoV-2 assay performance (S3 Table). Other endo- or exogenous substances, except ≥ 2.5% (w/v) mucin, had no negative effects on the amplification of the target genes nor the sample processing control. From the common laboratory disinfectants, isopropyl alcohol had a discernible interfering effect by inducing a failed result due to an abnormally low signal level.

Reproducibility of the GenomEra SARS-CoV-2 assay was confirmed at three sites using identical samples, one negative and one low positive (1.5 x LOD) sample, tested on five consecutive days. All reproducibility runs returned expected results at all sites and the SPC was positive in all runs. Sample stability in different transport media was evaluated using original untreated patient samples and pretreated samples. In both cases, immediate testing is recommended, but untreated samples can be preserved at +4°C for several days, and even storage at RT is tolerated (Table 3). When stored at RT sample testing needs to be performed within 48 h when eSwab is used. Storage of heat pretreated samples is not recommended for over 3h even at +4°C when using eSwab (Table 3). No major stability issues were observed in untreated sample storage up to 96 h in either +4°C or RT when UTM, saline or PBS was used as the storage media. In this group, pretreated samples also returned no erroneous results after 8 h of storage in either +4°C or RT.

**Table 3:** Sample stability in various transport media for untreated and pretreated samples used for the GenomEra SARS-CoV-2 assay.

**Untreated samples**								
	**UTM**		**eSwab**		**Saline**		**PBS**	
	Storage	Storage	Storage	Storage	Storage	Storage	Storage	Storage
Time (h)	(+4°C)	(RT)	(+4°C)	(RT)	(+4°C)	(RT)	(+4°C)	(RT)
0	2/2		2/2		2/2		2/2	
16	2/2	2/2	2/2	2/2	2/2	2/2	2/2	2/2
48	2/2	2/2	2/2	1/2	2/2	2/2	2/2	2/2
96	2/2	2/2	2/2	1/2	2/2	2/2	2/2	2/2
**Pretreated samples**								
	**UTM**		**eSwab**		**Saline**		**PBS**	
	Storage	Storage	Storage	Storage	Storage	Storage	Storage	Storage
Time (h)	(+4°C)	(RT)	(+4°C)	(RT)	(+4°C)	(RT)	(+4°C)	(RT)
0	2/2		2/2		2/2		2/2	
3	2/2	2/2	2/2	1/2	2/2	2/2	2/2	2/2
8	2/2	2/2	1/2	0/2	2/2	2/2	2/2	2/2

### Clinical performance

The GenomEra SARS-CoV-2 assay was evaluated retrospectively at four sites with 274 patient specimens preanalyzed for SARS-CoV-2 using one SOC comparator method and confirmed by an additional SOC method, if a discrepant result occurred. Samples were either fresh (184) or frozen (90) and collected into different transport media (S4 Table). Specimen collection media included UTM (73), eSwab (136), and saline (40). For 25 samples, the sample matrix was compatible but unknown (S4 Table). These 25 unidentified media were determined to be either saline or PBS, but the exact matrix could not be specified. During the GenomEra SARS-CoV-2 assay evaluation, 35 samples were permanently discarded from the study due to various reasons, and those were not included in the 274 patient specimens used for final assay evaluation. Causes for sample rejection were, over 72 h storage at 4°C (22), lack of result from both SOC methods (1), incompatible sample matrix (9), and insufficient number of samples (4) in tested matrix, PBS, preventing the assay validation.

Combined comparison to all SOC methods showed no false positive results (98/98) with the GenomEra SARS-CoV-2 test (Tables 4 and 5). Of the 10 false negatives (164/174) obtained using the GenomEra SARS-CoV-2 assay, 9 came from Site 3 using in-house RdRp gene RT-PCR. All 9 samples were detected with C_T_ values ranging from 31.2 to 38.2 with the primary and secondary SOC methods and were confirmed as positive after analysis with the Xpert Xpress SARS-CoV-2 secondary method. The final false negative was detected at Site 2 using in-house Envp gene assay, and the sample was confirmed positive also with the duplex RdRp in-house assay. From the 274 samples, 0.7% (2) were deemed inconclusive. In this category, the PCR run failed (1) or showed inhibition (1). Seventeen specimens (2 fresh and 15 frozen) were reported as negative with GenomEra SARS-CoV-2 test and positive with the primary SOC method. These samples (2 UTM, 4 saline, and 11 eSwab) were re-analyzed with the secondary SOC method, and all were confirmed as negative. During the course of the study, 16 instances of single-target positive results were recorded. In 12 cases, only RdRp amplification was detected (C_T_ 29.3–37.5) and in 4 cases only Envp was detected (C_T_ 26.2–35.0). In all cases, the tested samples were confirmed as positive. Of the 16 results, 6 were included in the final performance evaluation, 5 did not meet inclusion criteria and the remaining 5 instances were results from reanalysis.

**Table 4:** Agreement of the GenomEra SARS-CoV-2 test with SOC RT-PCR tests from fresh and frozen samples.

Samples		No. results (GenomEra vs. SOC)							
	n	TP^a^	TN	FP	FN	PPA %^b^		NPA %^c^	
**Fresh**	182	37	136	0	9	80.4	(66.1–90.6)	100	(97.3–100)
**Frozen**	90	61	28	0	1	98.4	(91.3–100)	100	(87.7–100)
**TOTAL** ^d^	272	98	164	0	10	90.7	(83.6–95.5)	100	(97.8–100)

^a^TP, true positive; TN, true negative; FP, False positive; FN, false negative.

^b^PPA, positive percent agreement {[Pos/Pos / (Pos/Pos + Neg/Pos)] x 100}.

^c^NPA, negative percent agreement {[Neg/Neg / (Neg/Neg + Pos/Neg)] x 100}.

^d^Two samples remained inconclusive; failed (1) or showed PCR inhibition (1).

**Table 5:** Transport media dependent agreement of the GenomEra SARS-CoV-2 test and SOC RT-PCR tests.

Samples		No. results (GenomEra vs. SOC)							
	n	TP	TN	FP	FN	PPA %		NPA %	
**eSwab**	136	51	81	0	4	92.7	(82.4–98.0)	100	(95.6–100)
**UTM**	71	16	54	0	1	94.1	(71.3–99.9)	100	(93.4–100)
**saline**	40	30	5	0	5	85.7	(69.7–95.2)	100	(47.8–100)
**unidentified**	25	1	24	0	0	100	(2.5–100)	100	(85.8–100)

**Fig 1:**
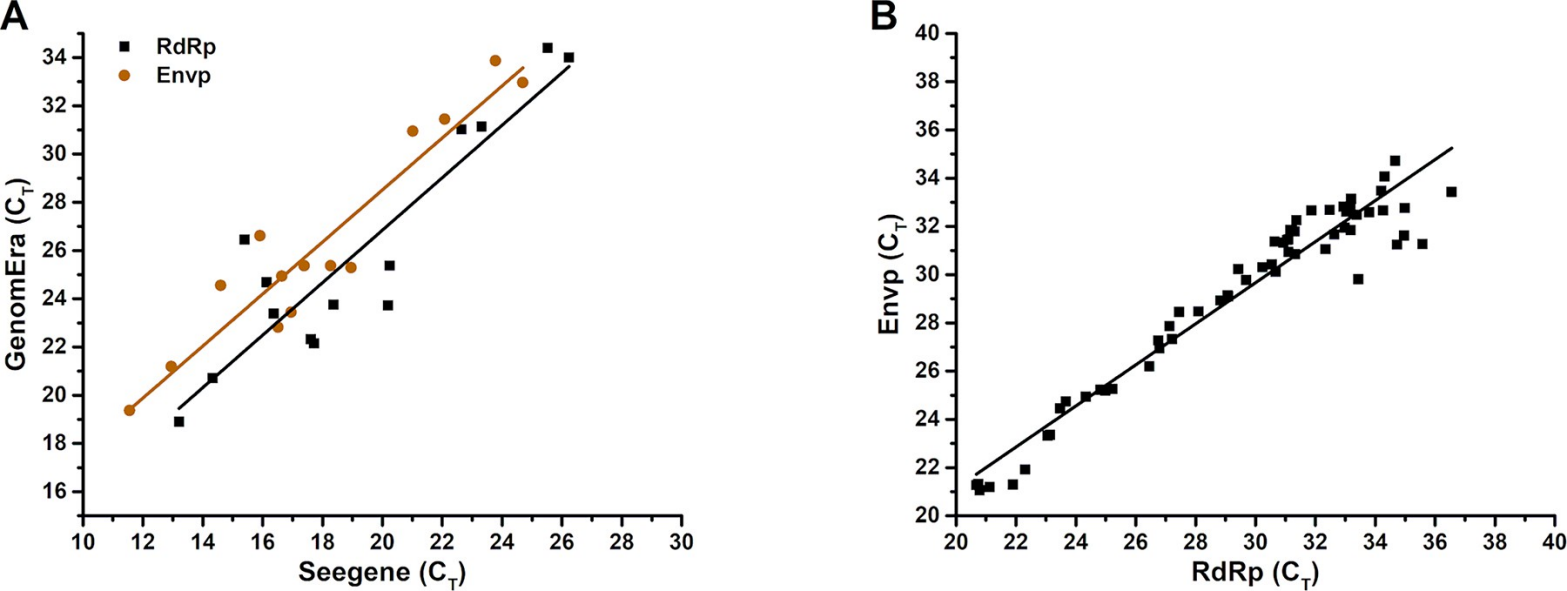
Linear correlations between the C_T_ values obtained using Seegene Allplex 2019-nCoV and GenomEra SARS-CoV-2 assays. (A) Fourteen positive samples were assayed using Seegene Allplex 2019-nCoV and GenomEra SARS-CoV-2 assays at Site 1, and the results were compared. Linear correlations were 0.91 and 0.94 for RdRp and Envp, respectively. (B) A linear correlation of 0.97 comparing RdRp and Envp was derived from 63 assay runs from 43 unique samples and 20 dual-runs using GenomEra SARS-CoV-2 assay.

C_T_ value correlation between the SOC method and GenomEra SARS-CoV-2 test was performed at Site 1 using the Seegene Allplex 2019-nCoV Assay. This method was selected to enable both Envp and RdRp comparison. A total of 14 samples gave positive results in both assays for both target genes (Fig 1A). In addition, 10 positive results were obtained only with Allplex. From these positive results, 3 were reported positive in the case of one or both Envp and RdRp, and an additional 7 for N gene only. All 10 were confirmed as false positives after secondary SOC analysis (Cepheid Xpert Xpress SARS-CoV-2). Linear correlation (Pearson’s r) between the C_T_ values obtained using GenomEra SARS-CoV-2 and Seegene Allplex 2019-nCoV assays were, 0.91 and 0.94 regarding RdRp and Envp, respectively (Fig 1A). Intra assay correlation, Pearson’s r between RdRp and Envp, was analyzed from the results obtained from Site 3 (Fig 1B). A total of 63 runs with both RdRp and Envp amplification detected from 43 unique positive samples were included in the analysis. Of the 43 samples, 20 were analyzed twice. Linear correlation between C_T_ values obtained from RdRp and Envp was 0.97.

## Discussion

We have presented here the analytical and clinical performance of the GenomEra SARS-CoV-2 Assay Kit, an easy-to-use RT-PCR test for the detection of SARS-CoV-2 Envp and RdRp genes. The GenomEra SARS-CoV-2 assay generates results for four samples in about 70 min, without RNA extraction steps. The GenomEra SARS-CoV-2 assay performed with the GenomEra CDX system is not a point-of-care test, but it requires only minimal laboratory resources and equipment. This enables use in centralized settings, providing strategic testing flexibility, or in decentralized settings to provide testing to meet smaller local needs. Up to eight analyzers can be linked to a single workstation, enabling testing for roughly 200 samples during a normal workday.

We found that the GenomEra SARS-CoV-2 assay did not produce false positive results in comparison to the used reference methods, which indicates excellent specificity and strong reliability of positive results. In addition, 8/10 of the false negative results from Site 3 were later confirmed not to be from randomly enrolled samples, but instead chosen with an intention to challenge the performance of GenomEra SARS-CoV-2 assay by testing samples with higher C_T_ values. Therefore, the C_T_ values of samples did not fully represent a natural distribution, introducing negative bias to the clinical performance values which resulted in a slightly lower PPA value than otherwise expected from a truly random data set. Nevertheless, the GenomEra SARS-CoV-2 assay failed to detect the several otherwise confirmed low positive samples highlighting sensitivity as one key limitation when compared to the SOC reference methods. The overall PPA of the assay was 90.7% and NPA 100%. Although the sensitivity of the assay may not equal that of methods utilizing RNA extraction, frequent sampling and timely testing have shown more clinical relevance in the fight against the Covid-19 pandemic [15].

According to our findings, eSwab exhibited the best analytical sensitivity among the tested transport media with a LOD of 8 IU/reaction, 239 IU/mL in the pretreated sample and 5012 IU/mL in the original sample. PBS, with the lowest analytical sensitivity of the group, had a nine-fold difference in LOD compared to eSwab. Furthermore, we were not able assess the clinical performance of PBS since we only encountered 4 samples in this media. The analytical performance data suggests eSwab should be used if available, with UTM being the second best option. From these matrices, eSwab showed the lowest sample stability, which was indicated only with storage in RT after 16 hours. Especially after heat treatment of samples in eSwab, the analysis should be performed without delay. No stability issues were observed with untreated samples in any media up to 96 hours when samples were stored at +4°C, enabling test repetition upon need. During the sample preparation process, the original samples are diluted over 500-fold, which significantly dilutes the potentially interfering compounds found in the samples, thus decreasing the possibility for PCR inhibition. Overall, we determined the assay to be generally robust against inhibition when tested with potentially interfering substances even at high concentrations. This all comes with a small sensitivity tradeoff, for not potentially detecting weak positive samples with low virus counts. Thus, the GenomEra SARS-CoV-2 assay might not be suitable for screening non-symptomatic patients, as the potentially lower viral load in these cases might cause false negative results [24–26]. In acute SARS-CoV-2 infection, the virus amount is typically extremely high and thus the assay is not usually limited by the test sensitivity [23,24]. Also, the transmissibility of COVID-19 positive patients depends on the viral load, highlighting the importance of detecting the individuals with high potential of mediating SARS-CoV-2 infection [24–26].

As vaccination efforts worldwide are progressing and new more transmissible and potentially vaccine-evasive viral variants arise, reliable on-demand testing for acute cases remains relevant into the foreseeable future to enable swift clinical and epidemiological actions. Using *in silico* analyses on available SARS-CoV-2 sequences as of January 2022, we have shown that the overall clinical performance of the GenomEra SARS-CoV-2 Assay is not easily hindered by the emergence of novel variants, the most recent being the Delta and Omicron variants. Other SARS-CoV-2 strains are also expected to be detected by the assay, taking into account the low prevalence of single nucleotide mutations in either Envp or RdRp and the lack of dual mutations in both target regions. Additionally, our findings suggest that the assay can generate reliable positive results with only one positive target. The reliability of single-target positive results is supported by our observation of 16 such instances with the GenomEra SARS-CoV-2 Assay from 11 different samples, which were all confirmed as positive with the SOC in-house RdRp assay. Because the chance of future mutations occurring in both target regions is considerably low, the assay will likely remain a reliable tool even in the rapidly changing field. The GenomEra SARS-CoV-2 assay needs no extensive laboratory equipment, and thus can provide an effective option to be used in e.g. acute-care hospitals in high-prevalence settings or more isolated locations, where only small testing capacity is needed. In these settings rapid results can enable fast protective actions and immediate targeted clinical treatment.

## Supporting information

S1 Table Reference RT-PCR methods for SARS-CoV-2 detection used in comparison to GenomEra SARS-CoV-2 test. Testing methods for SARS-CoV-2 detection used in each participating site at the time of the study.
(PDF)

S2 Table Microorganisms tested with the GenomEra SARS-CoV-2 assay for the study of analytical specificity. Various microorganisms of bacterial and viral origin in differing concentrations were tested with the GenomEra SARS-CoV-2 assay to assess the specificity of the assay. All microorganisms tested were originally obtained and isolated from clinical samples.
(PDF)

S3 Table Potentially interfering substances tested with the GenomEra SARS-CoV-2 assay. Assay robustness against interference was assessed by introducing various substances into the sample media and testing each one separately.
(PDF)

S4 Table Specifications and sites for the samples tested with the GenomEra SARS-CoV-2 assay. The assay evaluation was conducted in four clinical institutions and incorporated fresh and frozen clinical samples gathered mostly in eSwab, but also in UTM, saline and other unspecified sample media.
(PDF)
