# Supplementary material for: Multicenter evaluation of the GenomEra SARS-CoV-2 assay kit
Source: PLoS One. 2022 Nov 28;17(11):e0277925. doi: 10.1371/journal.pone.0277925 (PMC9704634; doi:10.1371/journal.pone.0277925)
Supplement: S1 Table — Testing methods for SARS-CoV-2 detection used in each participating site at the time of the study. (PDF) [file pone.0277925.s001.pdf]

S1 Table. Reference RT-PCR methods for SARS-CoV-2 detection used in comparison to GenomEra SARS-CoV-2 test. Testing methods for SARS-CoV-2 detection used in each participating site at the time of the study.

Evaluation Site	Name	Regulatory status	Target(s)
**Site 1, Fimlab, Tampere, Finland**			
Method 1	Abbott RealTime SARS-CoV-2	FDA EUA	RdRp, N2
Method 2	Seegene Allplex™ 2019-nCoV Assay	CE Mark	RdRp, Envp, N2
Method 3	Cepheid Xpert Xpress SARS-CoV-2	FDA EUA	Envp, N2
**Site 2, THL[a], Helsinki, Finland**			
Method 1	E gene in-house	WHO[b]	Envp
Method 2	Duplex RdRp in-house	WHO[c]	RdRp (IP2 and IP4)
**Site 3, Umeå University, Umeå, Sweden**			
Method 1	RdRp gene in-house	WHO	RdRp
Method 2	Cepheid Xpert Xpress SARS-CoV-2	FDA EUA	Envp, N2
**Site 4, TYKS[d], Turku, Finland**			
Method 1	Cepheid Xpert Xpress SARS-CoV-2	FDA EUA	Envp, N2
Method 2	Duplex E and RdRP gene in-house	WHO	RdRp, Envp

[a] Finnish Institute for Health and Welfare.
[b] Protocol recommended by the World Health Organization (WHO), first evaluated in the Charité Virology, Berlin, Germany.
[c] Protocol recommended by the WHO, first evaluated in the Institut Pasteur, Paris, France.
[d] Turku University Hospital
