# Supplementary material for: Multicenter evaluation of the GenomEra SARS-CoV-2 assay kit
Source: PLoS One. 2022 Nov 28;17(11):e0277925. doi: 10.1371/journal.pone.0277925 (PMC9704634; doi:10.1371/journal.pone.0277925)
Supplement: S2 Table — Various microorganisms of bacterial and viral origin in differing concentrations were tested with the GenomEra SARS-CoV-2 assay to assess the specificity of the assay. All microorganisms tested were originally obtained and isolated from clinical samples. (PDF) [file pone.0277925.s002.pdf]

S2 Table. Microorganisms tested with the GenomEra SARS-CoV-2 assay for the study of analytical specificity. Various microorganisms of bacterial and viral origin in differing concentrations were tested with the GenomEra SARS-CoV-2 assay to assess the specificity of the assay. All microorganisms tested were originally obtained and isolated from clinical samples.

Tested microorganism	Sample type	Sample concentration	Test result
Coronavirus 229E	Virus	Ct values 20.64–27.24[a]	Negative
Coronavirus HKU1	Virus	Ct values 17.58–24.95[a]	Negative
Coronavirus NL63	Virus	Ct values 20.83–23.38[a]	Negative
Coronavirus OC43	Virus	Ct values 20.19–26.65[a]	Negative
Influenza A(H1)pdm09	Virus	Ct values 17.98 and 22.46	Negative
Influenza A(H3)	Virus	Ct values 19.21 and 20.64	Negative
Influenza B (Victoria)	Virus	Ct values 20.56 and 21.05	Negative
Influenza B (Yamagata)	Virus	Ct values 21.46 and 22.35	Negative
Respiratory syncytial virus	Virus	Ct values 22.02 and 22.30	Negative
Rhinovirus	Virus	Ct values 19.71 and 38.95	Negative
Adenovirus	Virus	Ct values 22.02 and 22.19	Negative
SARS-CoV	RNA	10^6 and 10^7 copies/mL	Negative
MERS-CoV	RNA	Ct 27.04	Negative
Enterovirus D68	RNA	Ct value 21.33	Negative
Human parechovirus	RNA	2×10^5 copies/mL	Negative
Metapneumovirus A1	RNA	Ct 32.70	Negative
Metapneumovirus A2	RNA	Ct 31.00	Negative
Parainfluenzavirus 1	RNA	Ct 28.60	Negative
Mycoplasma pneumoniae	DNA	Ct value 28.00	Negative
Pseudomonas aeruginosa	Bacteria	> 0.2 Abs600 nm	Negative
Streptococcus pneumoniae	Bacteria	> 0.2 Abs600 nm	Negative
Streptococcus pyogenes	Bacteria	> 0.2 Abs600 nm	Negative
Streptococcus salivarius	Bacteria	> 0.2 Abs600 nm	Negative

[a] Four samples with varying CT values.
