# Supplementary material for: Multicenter evaluation of the GenomEra SARS-CoV-2 assay kit
Source: PLoS One. 2022 Nov 28;17(11):e0277925. doi: 10.1371/journal.pone.0277925 (PMC9704634; doi:10.1371/journal.pone.0277925)
Supplement: S3 Table — Assay robustness against interference was assessed by introducing various substances into the sample media and testing each one separately. (PDF) [file pone.0277925.s003.pdf]

S3 Table. Potentially interfering substances tested with the GenomEra SARS-CoV-2 assay. Assay robustness against interference was assessed by introducing various substances into the sample media and testing each one separately.

Endogeneous substances	Active ingredient	Concentration[a]	Test result
Mucin	Mucin protein	2.5% (w/v)	No effect
Blood (human)	Blood (human)	2 % (v/v)	No effect
**Endogeneous substances**			
Nasal corticosteroid 1	Beclomethasone	5% (v/v)	No effect
Nasal corticosteroid 2	Fluticasone	5% (v/v)	No effect
Throat lozenge, oral anesthetic and analgesic	Benzocaine	5 mg/mL	No effect
Anti-viral drug	Oseltamivir	7.5 mg/mL	No effect
Antibiotic, nasal ointment	Mupirocin	10 mg/mL	No effect
Beta-adrenergic bronchodilator	Salbutamol	10 mg/mL	No effect
Nasal adrenergic receptor agonist	Oxymetazoline	5% (v/v)	No effect
**Laboratory disinfectants**			
Ethanol	Ethanol	2% (v/v)	No effect
Hypochlorite	Sodium hypochlorite	0.01% (v/v)	No effect
Aseptic disinfectant	Isopropyl alcohol	1.5% (v/v)	Failed[b]

[a] Concentration in the transport media.
[b] Failed due to abnormally low signal level.
