# Supplementary material for: Multicenter evaluation of the GenomEra SARS-CoV-2 assay kit
Source: PLoS One. 2022 Nov 28;17(11):e0277925. doi: 10.1371/journal.pone.0277925 (PMC9704634; doi:10.1371/journal.pone.0277925)
Supplement: S4 Table — The assay evaluation was conducted in four clinical institutions and incorporated fresh and frozen clinical samples gathered mostly in eSwab, but also in UTM, saline and other unspecified sample media. (PDF) [file pone.0277925.s004.pdf]

S4 Table. Specifications and sites for the samples tested with the GenomEra SARS-CoV-2 assay. The assay evaluation was conducted in four clinical institutions and incorporated fresh and frozen clinical samples gathered mostly in eSwab, but also in UTM, saline and other unspecified sample media.

**Evaluation Site**		**Site 1**	**Site 2**	**Site 3**	**Site 4**	**Total**
**Fresh Samples**		**95**	**42**	**47**	**0**	**184**
	eSwab	71	0	18	0	
	UTM	23	23	0	0	
	Saline	1	0	29	0	
	Other[a]	0	19	0	0	
**Frozen Samples**		**63**	**10**	**0**	**17**	**90**
	eSwab	47	0	0	0	
	UTM	6	4	0	17	
	Saline	10	0	0	0	
	Other	0	6	0	0	
**Total**		**158**	**52**	**47**	**17**	**274**
	eSwab	118	0	18	0	**136**
	UTM	29	27	0	17	**73**
	Saline	11	0	29	0	**40**
	Other	0	25	0	0	**25**

[a] Unknown clear RT-PCR compatible media, saline or PBS.
